# Thermodynamic pathway of lignocellulosic acetylation process

**DOI:** 10.1186/s13065-019-0593-8

**Published:** 2019-07-03

**Authors:** Jude Chinedu Onwuka, Edith Bolanle Agbaji, Victor Olatunji Ajibola, Friday Godwin Okibe

**Affiliations:** 1 Department of Chemistry, Federal University Lafia, PMB 146, Nasarawa, Nigeria; 2 Department of Chemistry, Ahmadu Bello University Zaria, Kaduna, Nigeria

**Keywords:** Lignocellulosics, Acetylation, Thermodynamics, ANOVA, Critical, Weight percent gain

## Abstract

The use of natural cellulosic fibers as materials in the reinforcements of polymer composites and sorption of oil from water, has directed more focus on acetylation than other known chemical modification methods. Cellulose can be modified by acetylation to provide a suitable and cost effective cellulose acetate which have high hydrophobic characteristics and are biodegradable. In this study, lignocellulosic samples—oil palm empty fruit bunch (OPEFB), pride of Barbados pods (POBP) and cocoa pods (CP)—with different compositions of lignin and hemicellulose, were acetylated using solvent free method. Effect of temperature on the acetylation of these samples at different reaction times were studied and used for the thermodynamic studies. Analysis of variance (ANOVA) was used to test the significance of temperature variation with weight percent gain (WPG) due to acetylation of the lignocellulosics at different reaction times. FTIR studies showed evidence of successful acetylation reaction. ANOVA test showed no statistical difference in the observed variation of WPG due to acetylation of all the lignocellulosic samples, with temperature at different reaction times. The best acetylating period for OPEFB, POBP and CP were 60, 30 and 90 min respectively. Acetylation of the lignocellulosic samples were found to occur by absorbing heat from the environment. Values of entropy changes were positive while Gibb’s free energy change values were negative except at operating temperature of 303 K. Thus, acetylation of these lignocellulosic samples were spontaneous except at 303 K. Acetylated POBP has the lowest heat capacity (0.82 kJ mol^−1^ K^−1^) compared to acetylated OPEFB (1.47 kJ mol^−1^ K^−1^) and CP (1.15 kJ mol^−1^ K^−1^). Low critical WPG showed that the mechanism of acetylating these materials were diffusion controlled. The critical temperatures of OPEFB, POBP and CP acetylation were found to be 282.6 K, 223.2 K and 260.5 K respectively. Thus, acetylation of these lignocellulosic samples were successful and found to be energy efficient.

## Introduction

Lignocellulose are natural fibers which contains lignins and hemicellulose that has to be removed in order to obtain pure cellulose. In Nigeria, agro-wastes are the main sources of lignocellulose and these are readily and cheaply available. Agricultural by-products can be considered polymeric composites made up primarily of cellulose, hemicellulose and lignin [9, 12]. Hydroxyl functional groups are abundantly available in all the three major chemical components of agro based materials which is responsible for their hydrophilicity and lack of dimensional stability [4]. Moreover due to the hydroxyl group located on surface of cellulose, the surface is hydrophilic. In order to decrease the hydrophilic characteristics of the fibers and improve the surface adhesion between the continuous and dispersed phases, chemical modifications of the cellulose are needed [5, 10].

Lignocellulose has a lot useful purposes such as being source of fossil fuels, biofuels, fossil based packaging material, biofuel gelling agent, paper production, reinforcement in polymers, sorbents for removal of pollutants from aqueous medium etc. Agricultural wastes such as oil palm empty fruit bunch *(Elaeis guineensis)*, pride of Barbados (*Delonix regia*), and cocoa (*Theobroma cacao*) pods are very abundant in different parts of Nigeria.

Depending on the use of the agro waste lignocellulose, the surface structure of the lignocellulose may be modified. In packaging polymer material, it is important to have strong mechanical properties and to provide good control of mass transfer between food and the environment [21]. Hydrophobicity (oleophilicity) is one of the major determinants of sorbents properties influencing the effectiveness of oil sorption in the presence of water. The effectiveness of the sorbents in saturated environments would be enhanced if the density of the hydroxyl functionality is decreased [4].

Acetylation is one of the most commonly used modification methods. In acetylation reactions, the hydroxyl (OH) group of cellulose is substituted with acetyl (CH_3_CO) group; therefore the hydrophilic property is modified to be more hydrophobic. Meanwhile moderate acetylation does not change the original crystalline structure of cellulose, so the desired properties are also preserved [13, 18]. Acetic anhydride is commonly used as an acetylating agent reacting with free hydroxyl groups. In the acetylation of natural fibers, the product obtained contains acetyl groups bonded to the hydroxyl (OH) sites in lignocellulosic cell wall [7]. Due to the reported difference in the reactivity of hydroxyl functional groups of lignin, hemicellulose and cellulose, it is important to study the mechanism of acetylating the lignocellulosic materials because it is expected that the amount of lignin and hemicellulose will also affect the process as well.

This study investigates the thermodynamic nature of acetylating lignocellulosic samples from common agricultural residues so as to determine the mechanism and conditions for spontaneity of the process. This research will also aid in determining the minimum temperature required for acetylation and the most suitable acetylating period for each of the agricultural residues.

## Materials and methods

### Sample collection, identification and preparation

The lignocellulosic samples; oil palm empty fruit bunch (OPEFB) and cocoa pods (CP) were obtained from local farms at Anambra State while pride of Barbados pods (POBP) was collected from the premises of National Research Institute for Chemical Technology (NARICT), Zaria. The collected oil palm *(Elaeis guineensis)* empty fruit bunch, pride of Barbados (*Delonix regia*) pods, and cocoa (*Theobroma cacao*) pods were identified by Mr Namadi Sanusi in the Herbarium of the Department of Botany, Ahmadu Bello University Zaria—Nigeria. The voucher numbers of the identified OPEFB, CP and POBP were given as 0371, 2890 and 01917 respectively. The samples were cut, ground in a mortar and then, thoroughly washed with distilled water to remove foreign materials, and water soluble components. This allowed the samples to maintain balance. The washed samples were allowed to dry properly in sunlight for 12 h and then oven dried to a constant weight at 338 K.

After drying, the samples were sieved with laboratory sieves to obtain homogenous particle size using the BS410/1986 laboratory test sieve. A mechanical sieve shaker was used to separate the samples into the desired particle size (i.e., 425–625 µm).

### Acetylation of the lignocellulosic samples

The acetylation of the lignocellulosics under mild conditions, in the presence of *N*-bromosuccinimide (NBS), using acetic anhydride was carried out in a solvent free system as described by Sun et al. [19] and Onwuka et al. [16].

A portion (3 g) of the sample was placed in a 250 mL conical flask containing 60 mL of acetic anhydride and 0.6 g (1% of the solvent) *N*-bromosuccinimide (NBS). The reaction was allowed for 60 min at 303 K in a thermostated water bath. The reaction was repeated for 90, 120, 150 and 180 min at the same temperature. The variation of these reaction periods was considered at 323, 343 and 363 K temperatures with the same amounts of acetic anhydride and catalyst.

The flask was removed from the bath and the hot reagent was decanted. The sample was thoroughly washed with ethanol and acetone to remove unreacted acetic anhydride and acetic acid by-product. The products were oven-dried at 333 K for 16 h, and later cooled and stored in a plastic container prior to analysis. The extent or level of modification of the lignocellulosic samples due to acetylation was estimated using weight percent gain (WPG).

### Weight percent gain

The weight percent gain (WPG) was determined by gravimetric method as described by Thompson et al. [20] and Azeh et al. [2]. It was calculated on the basis of oven-dried unreacted fibers. The dried samples obtained were reweighed to determine the weight gain on the basis of initial oven dry measurements. WPG of the samples due to acetylation was calculated using the expression

1 $${\text{WPG }}\left( {{\% }} \right) = \left[ {\frac{\text{Weight gain}}{\text{Original weight}}} \right] \times 100$$

### Analysis of variance (ANOVA)

Two variance estimations are compared using ANOVA test: variance within group (the unsystematic variation or error in the data) and variance between groups (effects due to the experiment) [8].

In this study, independent variable (acetylating temperature) and dependent variable (weight percent gain) were compared using ANOVA.

### Fourier transform infra-red (FTIR) analysis

The FTIR spectra was recorded using Shimadzu-8400S Fourier Transform Infrared Spectrometer (FT-IR) over the spectra range of 4000–500 cm^−1^ with a resolution of 4 cm^−1^. This was carried out at the National Research Institute for Chemical Technology (NARICT) Zaria.

## Results and discussions

### Fourier transform infra-red (FTIR) spectra analysis

**Fig. 1:**
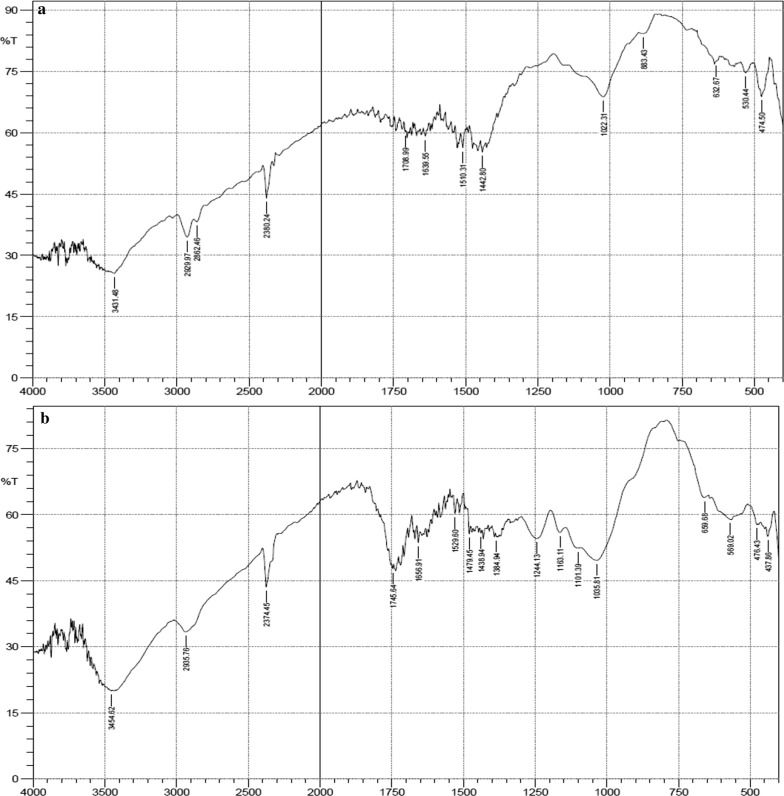
FTIR spectra of unacetylated (**a**) and acetylated OPEFB (**b**)

**Fig. 2:**
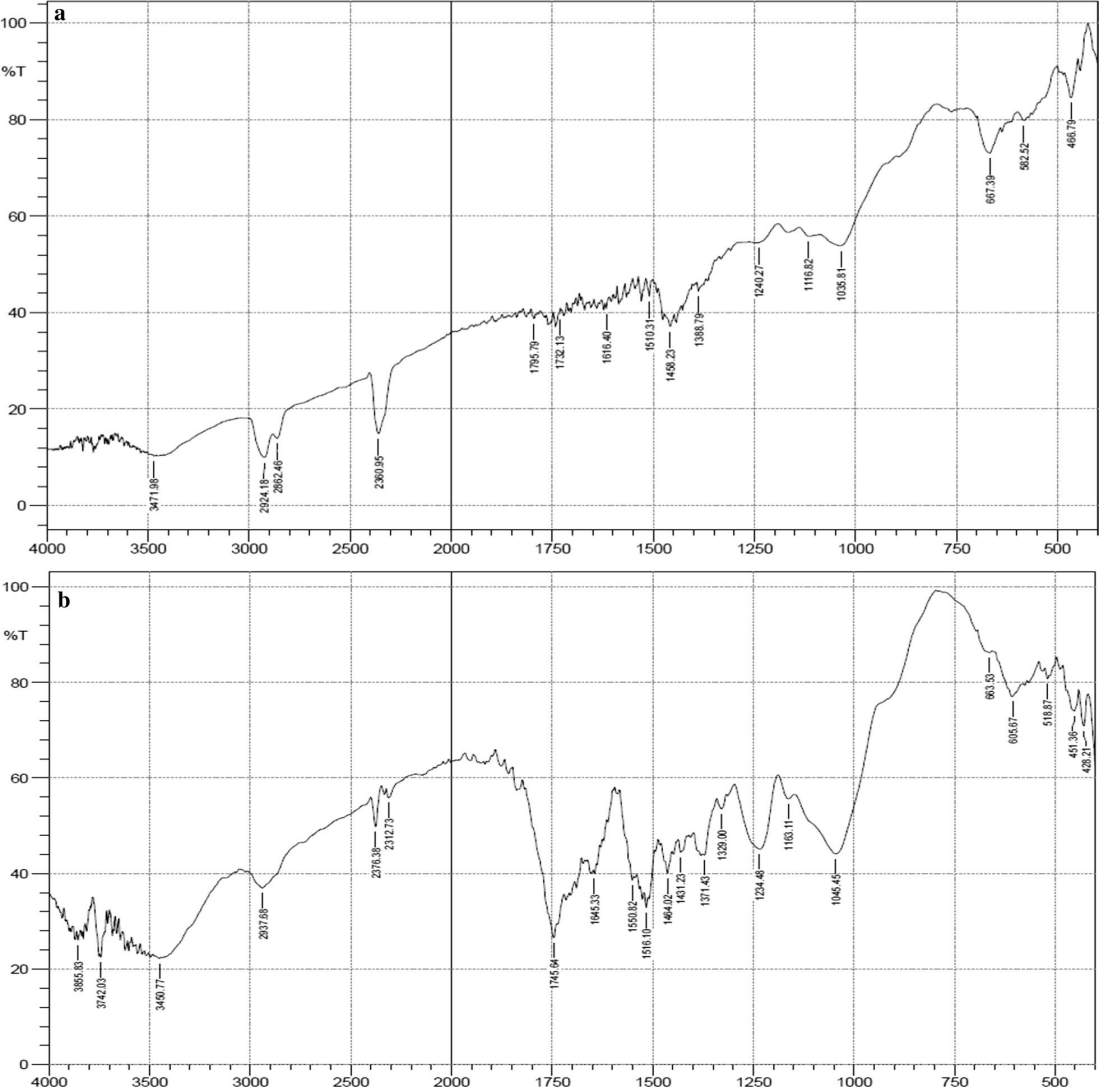
FTIR spectra of unacetylated (**a**) and acetylated POBP (**b**)

**Fig. 3:**
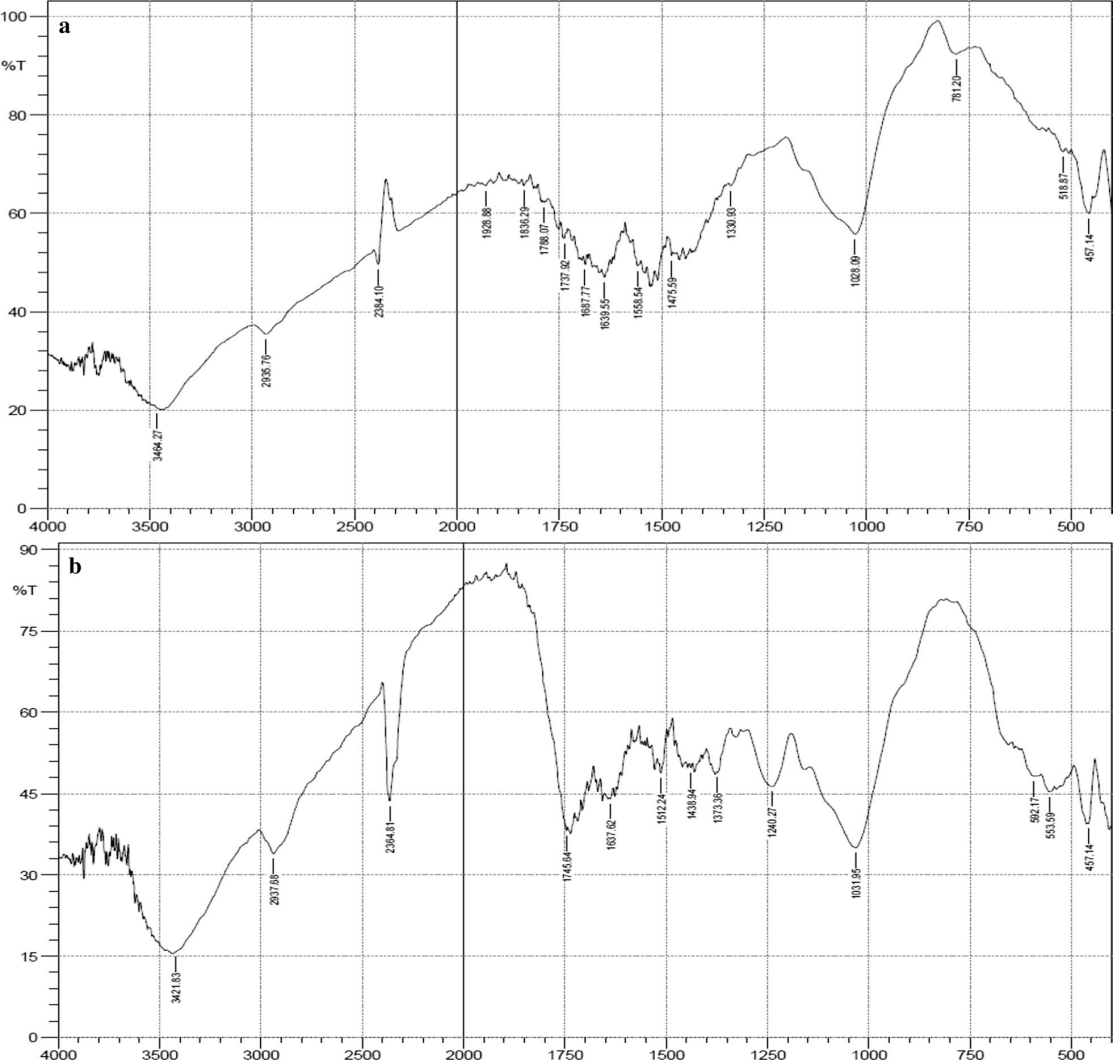
FTIR spectra of unacetylated (**a**) and acetylated CP (**b**)

Figures 1, 2 and 3 represents the IR spectra of unacetylated and acetylated OPEFB, POBP and CP respectively. The FTIR spectra showed that after acetylation, ester bands were shifted and enhanced at around 1745 cm^−1^ (carbonyl C=O stretching of ester), 1375 cm^−1^ (C–H in –O(C=O)–CH_3_), 1240 cm^−1^ (C–O stretching of acetyl group) and 1020 cm^−1^ (C–O stretching vibrations in cellulose) [1, 14, 15, 17]. Thus, confirming the successful acetylation of the lignocellulosic samples.

### Effect of temperature

**Fig. 4:**
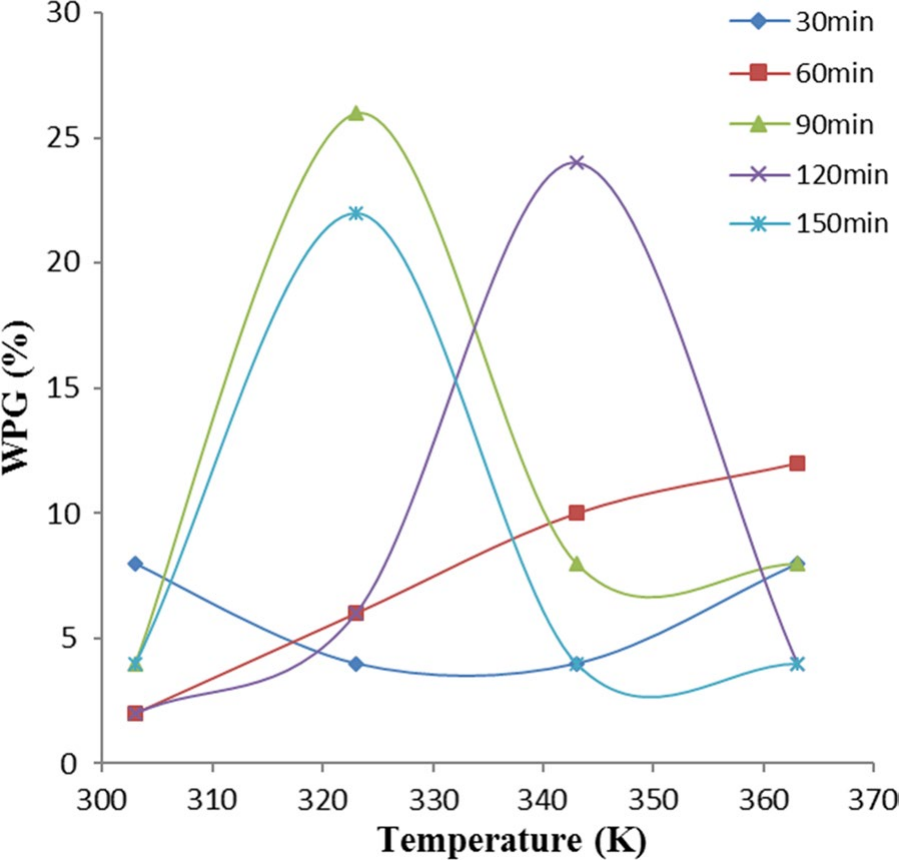
Effect of temperature on weight percent gain due to oil palm empty fruit bunch (OPEFB) acetylation

**Fig. 5:**
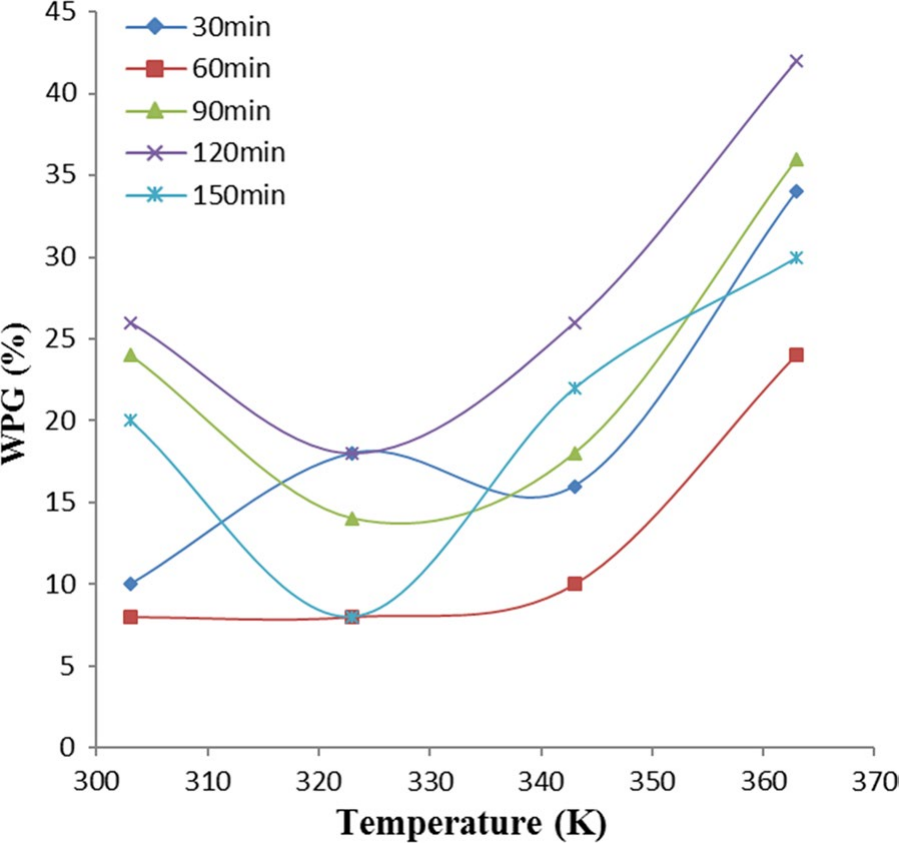
Effect of temperature on weight percent gain due to pride of barbados pod (POBP) acetylation

**Fig. 6:**
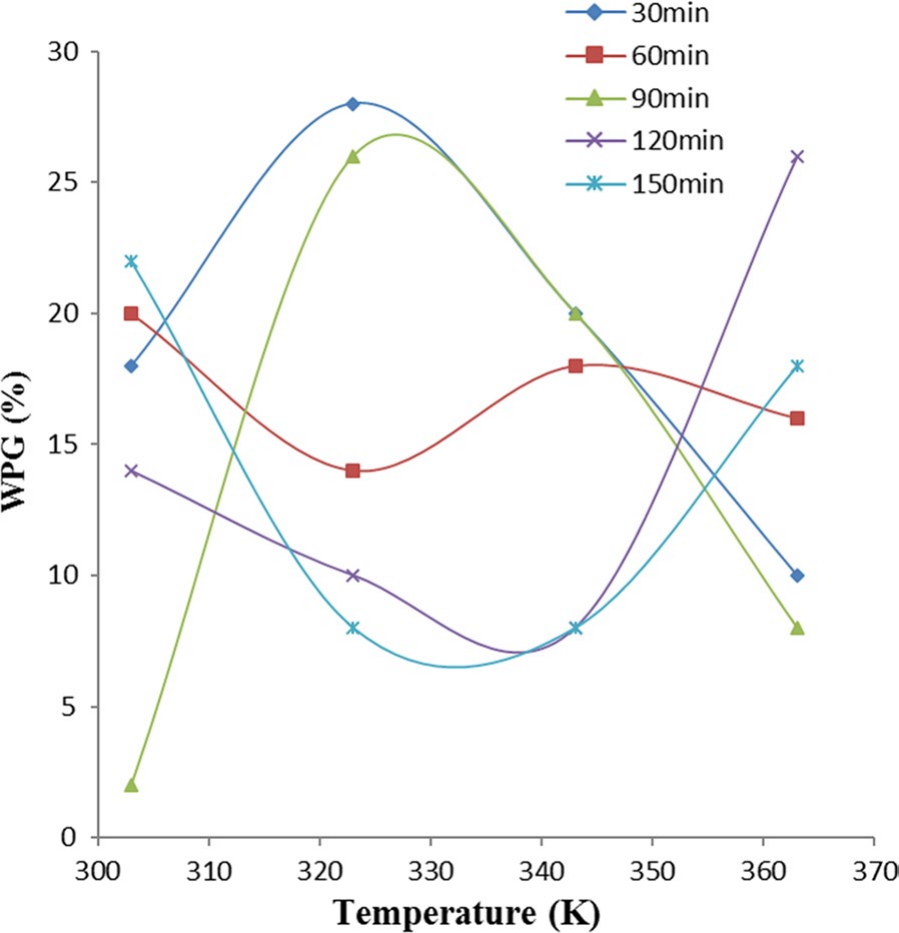
Effect of temperature on weight percent gain due to cocoa pod (CP) acetylation

Figures 4, 5 and 6 show the effect of temperature on weight percent gain (WPG) due to acetylation of OPEFB, POBP and CP, at different time intervals. Acetylation of each of the lignocellulosic samples did not show similar trend with temperature variation at different time intervals.

Figure 4 shows the effect of temperature on the WPG due to acetylation of OPEFB. In OPEFB acetylation at 60 min, there was continuous increase in WPG with increase in temperature. However, at 90, 150 and 120 min of acetylation, there was a sharp increase in WPG as their temperatures were increased to 323 K, 323 K and 343 K respectively, beyond which the WPG decreased. At 30 min, acetylation of OPEFB showed a decrease in WPG until the temperature was increased beyond 343 K.

Figure 5 revealed that at 90, 120, and 150 min acetylation of POBP, there was a decrease in WPG until the temperature was increased beyond 323 K. However, constant increase in WPG with increase in temperature was observed at 60 min acetylation period. At 30 min acetylation time, WPG follows no regular trend as the temperature was varied. Figure 5 also showed that the highest level of POBP acetylation at various temperatures was obtained at 120 min acetylation period.

It can be observed from Fig. 6 that acetylating CP at longer period (120 and 150 min), showed a decrease in WPG through a minimum until the temperature was increased beyond 343 K while acetylating at 30 and 90 min showed increase in WPG through a maximum until the temperature was increased above 323 K before WPG decreased constantly. WPG variation on 60 min acetylating period showed no regular trend.

The reasons for significant increase in acetylation with temperature increase, exhibited by some of the samples were probably due to the favourable effect of temperature on the compatibility of reaction ingredients and swellability of the cellulosic fibers [11]. In addition, the hydroxyl group of the cell wall polymers forms extensive hydrogen bonding networks within the matrix, and the reaction of the anhydride with hydroxyl group requires the breaking of a hydrogen bond [6]. During the acetylation process, the fiber swells as the reaction proceeds, requiring disruption of the hydrogen bonding network. In general, increasing temperature favoured breaking such hydrogen bonds, swelling the fibers, diffusing the esterifying agent and moving the reactant molecules, thus enhancing the reaction rate [20].

Furthermore, the increase and decrease in WPG observed, could probably be due to acetylation and de-acetylation mechanism [1]. During the increase in WPG at varied temperature, acetylation mechanism is possibly far exceeding de-acetylation while during decrease in WPG at varied temperature, de-acetylation mechanism is possibly far exceeding acetylation mechanism. The complex constituent (i.e., lignin, hemicellulose, holocellulose) nature of these samples could also be a possible reason. The difference in the composition nature of these samples is responsible for their different behaviours towards variation of temperature at different operating periods [1, 6].

### One-way ANOVA

In Figs. 4, 5 and 6, the variations of weight percent gain (WPG) due to acetylation of the samples, with temperature at various reaction times were shown. Analysis of variance (ANOVA) results presented in Table 1 showed that the observed variations of WPG as temperature was varied at different reaction times, have no significant/statistical difference.

**Table 1:** ANOVA for temperature effect on WPG of the samples at different time variation

Sample	Source of variation	SS	df	MS	F_cal_	F_crit_	P-value
OPEFB	Between groups	66	4	16.5	0.269902	3.055568	0.89277
	Within groups	917	15	61.13333			
	Total	983	19				
POBP	Between groups	510.8	4	127.7	1.448941	3.055568	0.266603
	Within groups	1322	15	88.13333			
	Total	1832.8	19				
CP	Between groups	79.2	4	19.8	0.333333	3.055568	0.851244
	Within groups	891	15	59.4			
	Total	970.2	19				

From the ANOVA results, we can conclude that null hypotheses were accepted at α = 0.05, because; *p* values are greater than α = 0.05. Another reason for the aforementioned conclusion could be based on the fact that F_cat_ < F_crit_, where F_cal_ for OPEFB, POBP and CP are 0.27, 1.45 and 0.33 respectively (Table 1).

Therefore, ANOVA results in Table 1 infer that the differences in the analyzed means of WPG due to acetylation of all the lignocellulosic samples, at operating temperatures of 303 K, 323 K, 343 K and 363 K at different reaction periods, were not enough to show that statistical/significant difference exist between them.

### Thermodynamics of acetylation

The relationship between temperature and weight percent gain (WPG) can be given as

2 $${ \ln }\,\,{\text{WPG}} = {\text{A}} - \frac{{\text{B}}}{{\text{T}}}$$

where ${\text{B}} = - \frac{{\Delta {\text{H}}}}{\text{R}}$ and *A* is the intercept. Thus, Eq. 2 is equivalent to the Arrhenius equation which is given by

3 $${ \ln }\,\,{\text{WPG}} = - {\frac{{{\Delta{\text{H}}}}}{{\text{RT}}}}$$

Infact Eq. 3 is the Arrhenius equation

4 $$\frac{{\text{dlnWPG}}}{{\text{dT}}} = {\frac{{{\Delta {{\text{H}}}}}}{{{{{\text{RT}}}^{2}} }}}$$

Integrating Eq. 4 is as shown below

5 $$\int\limits_{{{\text{WPG}}_{\text{o}} }}^{{{\text{WPG}}_{\text{T}} }} {{\text{dlnWPG}} = } \frac{{\Delta {\text{H}}}}{\text{R}}\int\limits_{{{\text{T}}_{ 0} }}^{\text{T}} {\frac{ 1}{{{\text{T}}^{ 2} }}} {\text{dT}}$$

Equation 5 gives

6 $$\left[ {{ \ln }\,{\text{WPG}}} \right]_{{{\text{WPG}}_{ 0} }}^{{{\text{WPG}}_{\text{T}} }} = - \frac{{\Delta {\text{H}}}}{\text{R}}\left[ {{\text{T}}^{ - 1} } \right]_{{{\text{T}}_{ 0} }}^{\text{T}}$$

7 $${ \ln }\left( {\frac{{{\text{WPG}}_{\text{T}} }}{{{\text{WPG}}_{ 0} }}} \right) = - \frac{{\Delta {\text{H}}}}{\text{RT}} + \frac{{\Delta {\text{H}}}}{{{\text{RT}}_{ 0} }}$$

8 $${ \ln }\,{\text{WPG}}_{\text{T}} = - \frac{{\Delta {\text{H}}}}{\text{RT}} + \frac{{\Delta {\text{H}}}}{{{\text{RT}}_{ 0} }} + { \ln }\,{\text{WPG}}_{ 0}$$

Equation 8 allows the plot of ln *WPG*_*T*_ versus *T*^−*1*^ such that $- \frac{{\Delta {\text{H}}}}{\text{R}}$ is the slope, intercept on *y*-axis gives $\frac{{\Delta {\text{H}}}}{{{\text{RT}}_{ 0} }}$ and intercept on *x-*axis gives *lnWPG*_*o*_. *∆H* is the heat of acetylation, *T*_*o*_ is the critical temperature of acetylation (below which the sample acetylation is not feasible), and *WPG*_*0*_ is the critical weight percent gain (below this value suggest surface adsorption mechanism while above it suggest diffusion mechanism) of the acetylated samples.

ANOVA result in Table 1 showed no significant difference in the variation of WPG due to acetylation of these samples, with temperature at various times. Thus, the linear equation with the best fit (i.e. the highest *R*^*2*^) within the acetylation time range studied, gives the best acetylating time for the sample and is also used as the equation for the thermodynamic plot of that sample using Eq. 8.

**Fig. 7:**
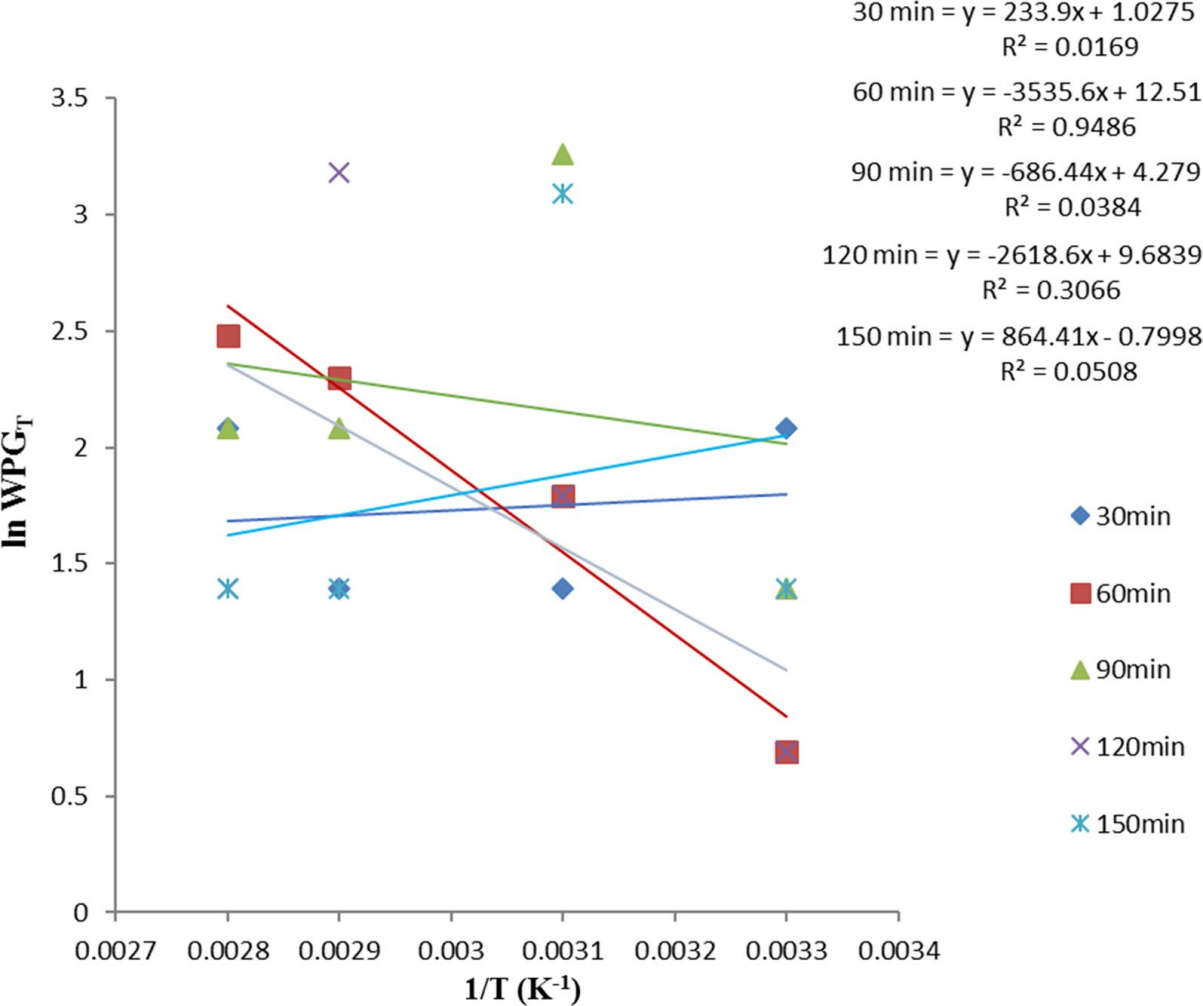
Thermodynamics plot for oil palm empty fruit bunch (OPEFB) acetylation using Eq. 8

**Fig. 8:**
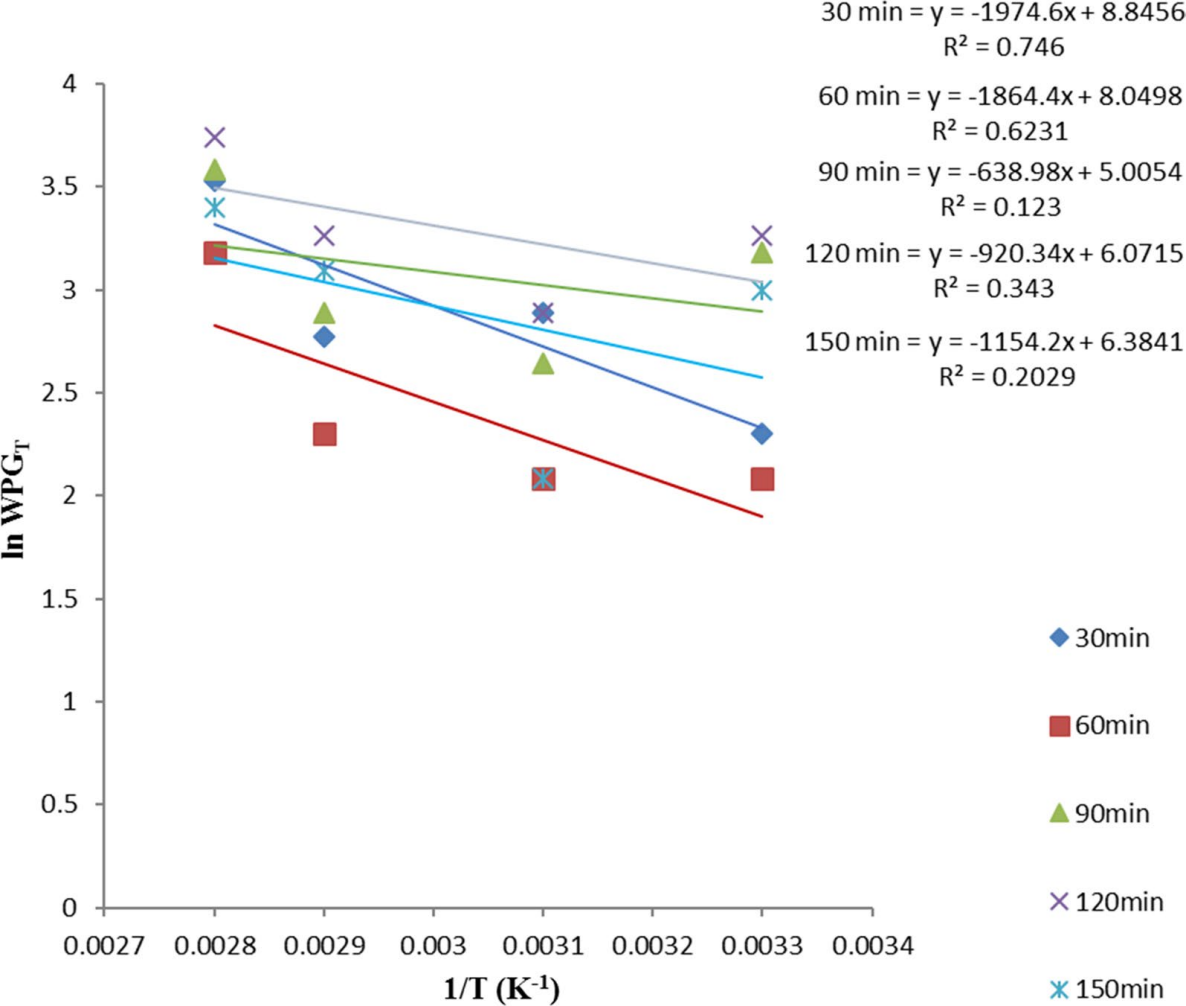
Thermodynamics plot for pride of barbados pods (POBPs) acetylation using Eq. 8

**Fig. 9:**
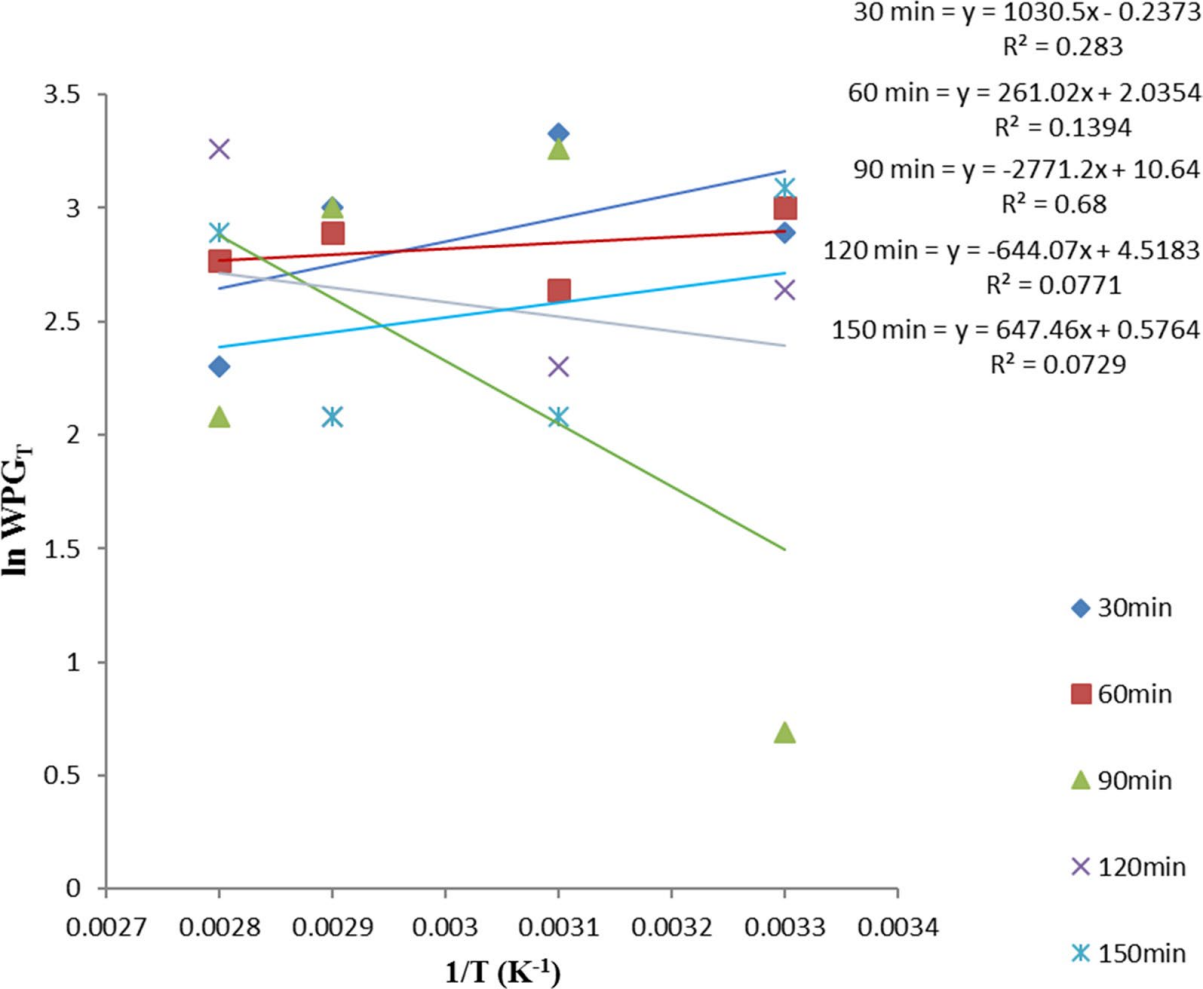
Thermodynamics plot for cocoa pod (CP) acetylation using Eq. 8

Figures 7, 8 and 9 showed that the best linear relationship for the thermodynamic plots of OPEFB, POBP and CP acetylation, was obtained at their 60, 30 and 90 min acetylating duration respectively. Thus,$y = - 3535x + 12.51$, $y = - 1974.6x + 8.8456$ and $y = - 2771.2x + 10.64$ represent the observed linear expression for thermodynamic plots of OPEFB, POBP and CP acetylation respectively.

Table 2 shows the thermodynamic data obtained from the plots using Eq. 8. From the value of the slope, the heats (enthalpies) of OPEFB, POBP, and CP acetylation were calculated to be 29.40 kJ mol^−1^, 16.42 kJ mol^−1^ and 23.04 kJ mol^−1^ respectively. From the intercept on the y-axis, the calculated critical temperatures of OPEFB, POBP and CP acetylation are 282.6 K (9.62 °C), 223.2 K (− 49.8 °C) and 260.5 K (− 12.55 °C) respectively. Then, from the intercept at the x-axis, the critical WPG due to acetylation for OPEFB, POBP and CP was found to be 0.75, 1.12, and 0.40% respectively.

**Table 2:** Parameters obtained from thermodynamics plots using Eq. 8

Sample	T_o_ (K)	WPG_o_	C_p_ (J mol^−1^ K^−1^)	∆H (J mol^−1^)	∆S (J mol^−1^ K^−1^)	∆G	Temp. (K)
OPEFB	282.60	0.75	1469.75	29395.00	93.90	− 4690.70	363
						− 2812.70	343
						− 934.70	323
						943.3*	303
POBP	223.20	1.12	820.84	16416.80	52.45	− 2623.2	363
						− 1573.55	343
						− 524.55	323
						523.65*	303
CP	260.45	0.40	1151.99	23039.80	73.60	− 3681.40	363
						− 2205.00	343
						− 733.80	323
						739.00*	303

*Acetylating temperature of the samples with positive change in Gibb’s free energy

Positive heat (enthalpy change) of acetylation implies that acetylation of these samples occurs by absorbing heat from the environment. The very low values of critical temperatures suggest that acetylation of these samples can take place easily at mild conditions. Thus, high critical temperatures would have suggested difficulty in acetylating the samples.

In thermodynamics, a critical point (or critical state) is the end point of a phase equilibrium curve [3]. On this basis, critical weight percent gain (WPG_o_) due to acetylation represents a value which gives information on the mechanism of the material acetylation. Values of WPG due to acetylation at a particular time (i.e., WPG_t_), which are above the critical WPG suggest diffusion mechanism while below it, suggest surface adsorption mechanism. Thus, the low critical WPG obtained due to acetylation suggests that diffusion mechanism played an important role in the acetylation of these samples. This further supports the suggestion by the kinetic studies of acetylation we earlier reported in Onwuka et al. [16], that diffusion mechanism was involved in acetylating pods of *Delonix regia* (i.e. pride of Barbados pods).

### Heat capacity, entropy and free energy of acetylation

The heat capacity (C_p_) of the acetylated samples at constant pressure was calculated using;

9 $$\Delta {\text{H}} = {\text{C}}_{\text{p}} \mathop \int \limits_{{{\text{T}}_{1} }}^{{{\text{T}}_{2} }} {\text{dT}} = {\text{C}}_{\text{p}} \left( {{\text{T}}_{2} - {\text{T}}_{1} } \right)$$

*C*_*p*_ represents the quantity of heat needed to raise the temperature of acetylation of the samples by one degree. *T*_*2*_ and *T*_*1*_ are the final and initial temperatures.

From the calculated heats (enthalpies) of OPEFB, POBP, and CP acetylation and change in temperature, the heat capacity (C_p_) values of OPEFB, POBP and CP acetylation are 1.47 kJ mol^−1^ K^−1^, 0.82 kJ mol^−1^ K^−1^ and 1.15 kJ mol^−1^ K^−1^ respectively as shown in Table 2. Thus, acetylated POBP has less heat content compared to the other two samples. This suggests possible occurrence of chemical reaction at room temperature.

The change in entropy of acetylation (∆S) can be obtained using the equation:

10 $$\Delta {\text{S}} = {\text{C}}_{\text{p}} \ln \frac{{{\text{T}}_{2} }}{{{\text{T}}_{1} }} + {\text{R ln}}\frac{{{\text{P}}_{1} }}{{{\text{P}}_{2} }}$$

where ∆S is the change in entropy, *T*_*1*_ and *T*_*2*_ are the initial and final operating temperatures and *P*_*1*_ and *P*_*2*_ are the initial and final operating pressures respectively. If the process is performed under the same pressure, the second term in right hand side of the equation becomes zero.

Table 2 shows that the values of change in entropy of the samples acetylation are positive (93.9 J mol^−1^ K^−1^ for OPEFB, 52.5 J mol^−1^ K^−1^ for POBP and 73.6 J mol^−1^ K^−1^ for CP), which suggests spontaneity in the acetylation process of these samples.

The free energy at different operating temperatures was calculated using

11 $$\Delta {\text{G}} = \Delta {\text{H}} - {\text{T}}\Delta {\text{S}}$$

It was found that for each of the samples, the value of free energy is positive at 303 K but as the temperature was increased to 323 K, 343 K and 363 K, the free energy value became negative. Thus, suggesting that though acetylation took place at low temperature (303 K), the process is not spontaneous because of positive free energy but at higher temperature it is spontaneous. This suggests that acetylation reaction proceeds spontaneously by absorbing heat from the environment (endothermic process). The thermodynamics of acetylation of these samples confirms their successful acetylation and is consistent with reports by Nwadiogbu et al. [14] who used extent of acetylation (not WPG) from Fourier transform infra-red (FTIR) technique, in the thermodynamic modelling.

## Conclusion

Thermodynamic modelling showed that acetylation process of these lignocellulosic samples are endothermic since enthalpy values are positive and the suitable acetylation period of the samples differs. The values of entropy and Gibb’s free energy change of the acetylation process of all the samples, suggests spontaneity in the acetylation of the samples at all temperatures except 303 K. Less quantity of heat was required to acetylate POBP compared to OPEFB and CP. Heat capacity values of the acetylated OPEFB, POBP and CP are 1.47, 0.82 and 1.15 kJ mol^−1^ K^−1^ respectively. All the lignocellulosic samples have very low critical WPG and temperature which suggests that diffusion mechanism responsible for their acetylation process and also, acetylation of these lignocellulosic samples can take place easily at mild conditions respectively. Thus, acetylation of lignocellulose is an energy efficient process.

## Data Availability

The raw data is available in the Ph.D. thesis titled “Modification and Characterization of Oil Palm Bunch, Pride of Barbados and Cocoa Pods as Sorbents for Crude Oil in Water” submitted to School of Postgraduate Studies, Ahmadu Bello University Zaria—Nigeria. The thesis is available on the University dissertation/thesis database.
